# Supplementary material for: A hypomorphic variant in EYS detected by genome-wide association study contributes toward retinitis pigmentosa
Source: Commun Biol. 2021 Jan 29;4:140. doi: 10.1038/s42003-021-01662-9 (PMC7846782; doi:10.1038/s42003-021-01662-9)
Supplement: Supplementary file 2 — Supplementary Information [file 42003_2021_1662_MOESM2_ESM.pdf]

A hypomorphic variant in *EYS* detected by genome-wide association study contributes toward retinitis pigmentosa

Nishiguchi KM et al.,

<u>SUPPLEMENTARY INFORMATION</u>

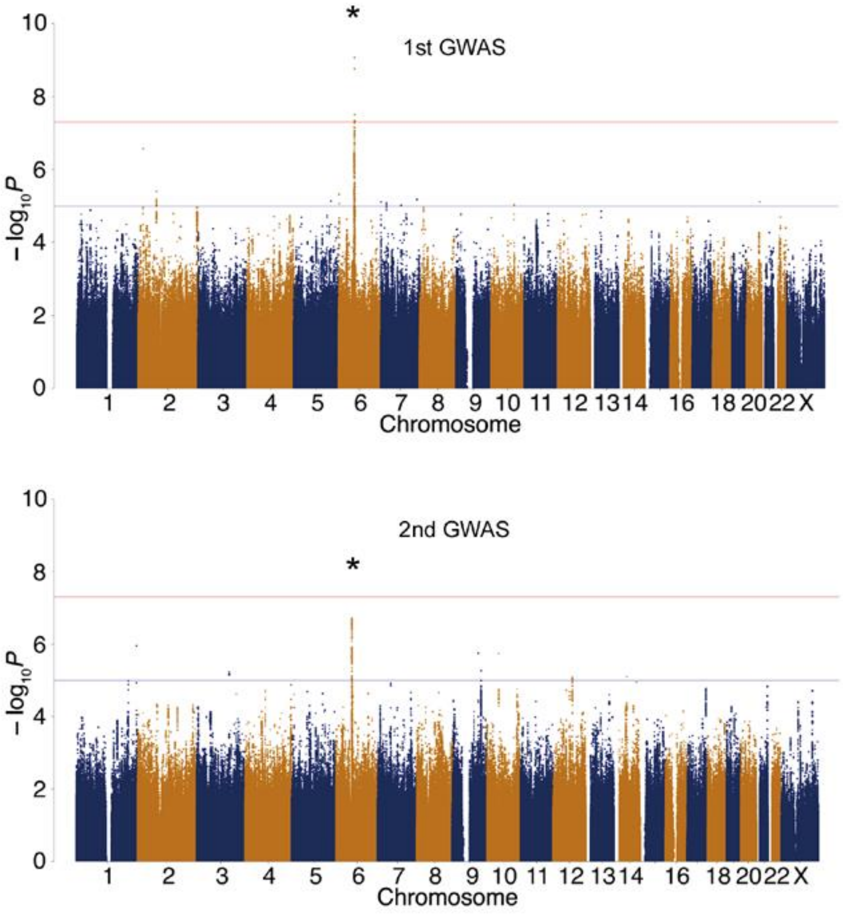

Supplementary Figure 1. Manhattan plots of 1st GWAS and 2nd GWAS

The *EYS* locus is labeled with an asterisk. GWAS, genome-wide association study. Genome-wide significance (P = 5.0 x10^{-8}) and possible significance (P = 1.0x10^{-5}) are marked with red and blue lines, respectively.

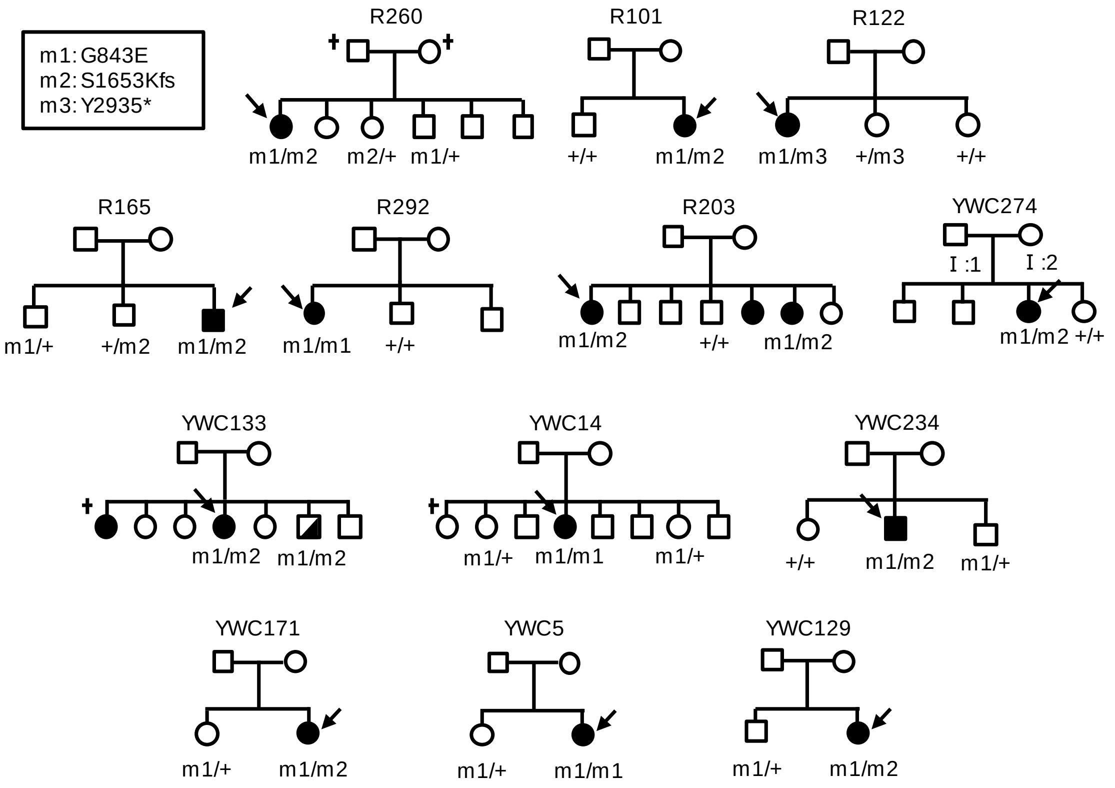

Supplementary Figure 2. Co-segregation of the G843E genotype with phenotype as an ARRP allele

The arrows indicate index patients. The half-filled symbol indicates an asymptomatic retinitis pigmentosa patient initially diagnosed as unaffected.

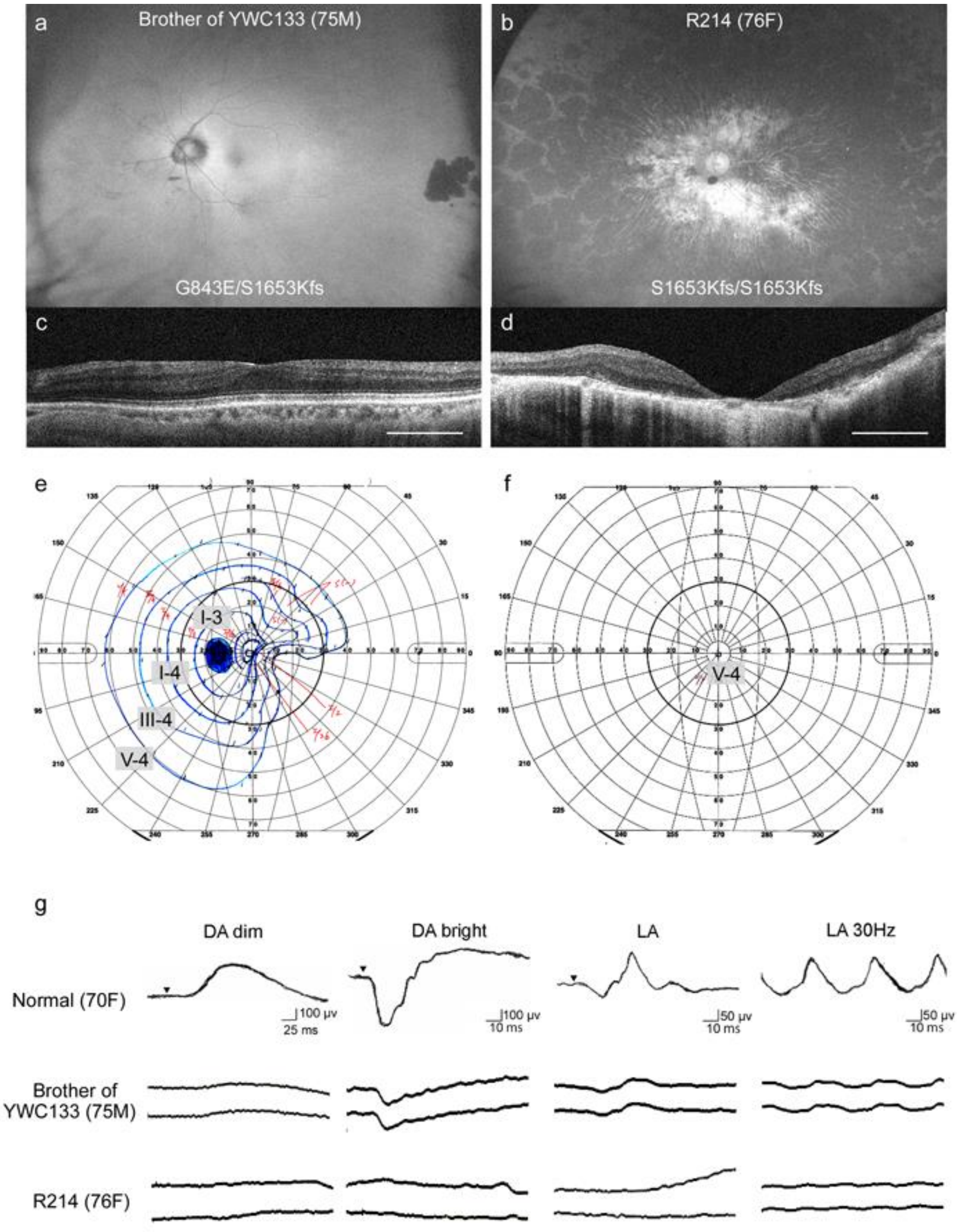

Supplementary Figure 3. Retinal degeneration in asymptomatic brother of YWC133

a-f, Representative data from the left eyes. Fundus autofluorescence (**a**,**b**), optical coherence

tomography (**c**,**d**), and Goldman perimetry (**e**,**f**) in a 75 years-old asymptomatic brother of YWC133 with G843E and S1653Kfs (**a**, **c**, **e**) and a 75 years-old female (R214) with homozygous S1653Kfs. The former had a normal vision (20/20) in both eyes whereas the latter had severely reduced acuity (20/400 OD and hand motion OS). Note abnormalities in fundus autofluorescence, optical coherence tomography, and Goldman perimetry are much milder in the brother of YWC133 compared to R214.

g. Electroretinogram. Electroretinogram recorded following a standard protocol outlined by the International Society for Clinical Electrophysiology of Vision showed severely reduced responses in both patients. DA, dark-adapted; LA, light-adapted.

Scale bar: 1.0 mm.

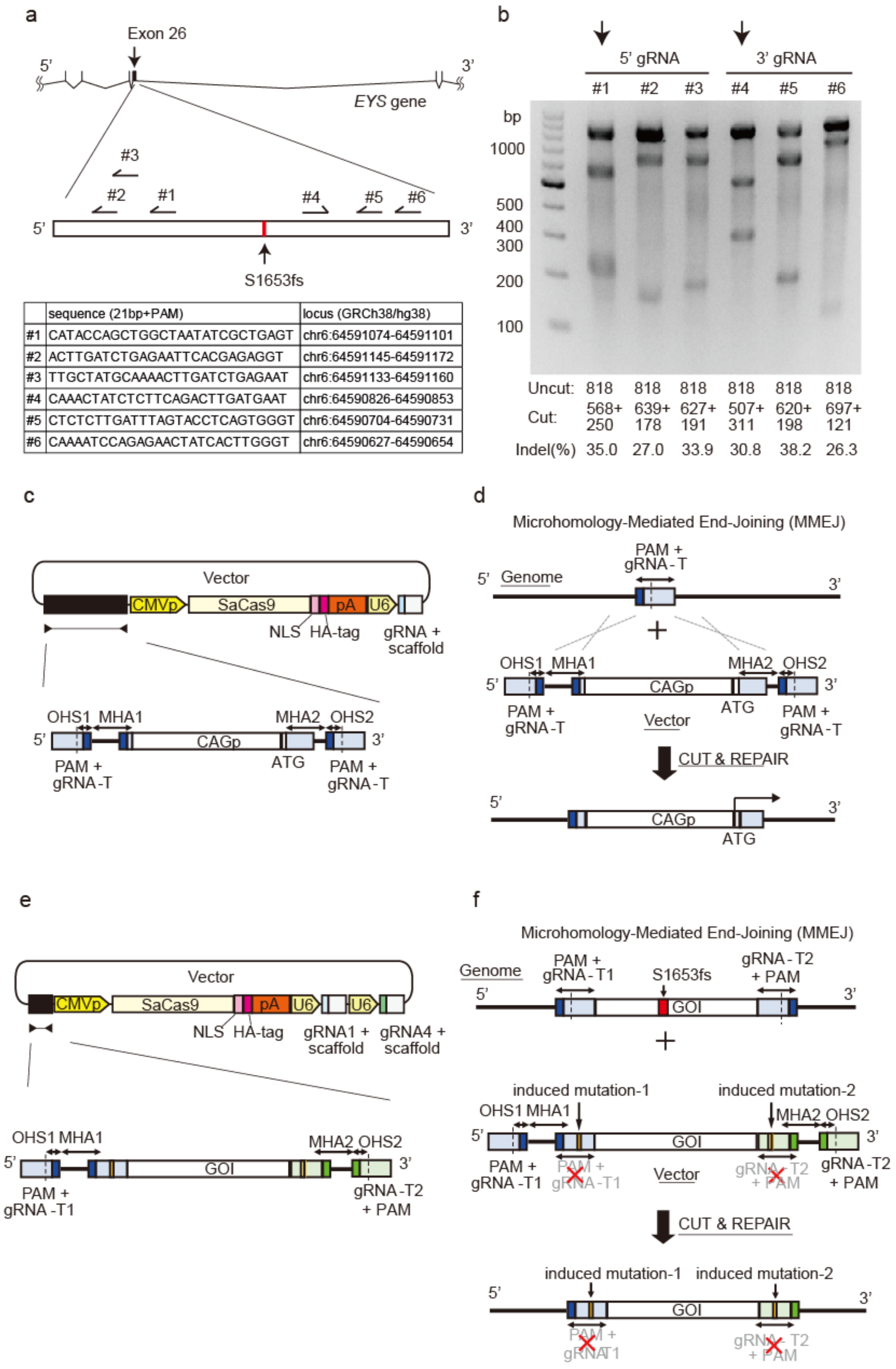

	sequence (21bp+PAM)	locus (GRCh38/hg38)
#1	CATACCAGCTGGCTAATATCGCTGAGT	chr6:64591074-64591101
#2	ACTTGATCTGAGAATTCACGAGAGGT	chr6:64591145-64591172
#3	TTGCTATGCAAAACTTGATCTGAGAAT	chr6:64591133-64591160
#4	CAAACTATCTCTTCAGACTTGATGAAT	chr6:64590826-64590853
#5	CTCTCTTGATTTAGTACCTCAGTGGGT	chr6:64590704-64590731
#6	CAAAATCCAGAGAACTATCACTTGGGT	chr6:64590627-64590654

Supplementary Figure 4. Genome editing strategy to insert CAG promoter or correct S1653Kfs mutation in lymphoblastoid cell lines (LCLs)

a. Design of targeting plasmid to insert a *CAG* promoter immediately upstream of the initiation codon of *EYS* gene through geno me editing. **b**. Illustration of the microhomology-mediated end-joining *CAG* promoter knock-in strategy. The DNA sequence of the LCL genome and plasmid vector were excised at the gRNA target sites (gRNA-T; dotted line) by two gRNAs and SaCas9. A *CAG* promoter was inserted into the genome using micro homology arms (MHA). **c**. Schematic map of gRNA designed inside exon 26 and list of their sequences. **d**. T7E1 assay for each gRNAs. Expected DNA size and quantified editing efficiency are displayed under the representative gel image. gRNA-1 and -4 were selected for the downstream mutation replacement genome editing experiment. **e**. Design of targeting plasmid to correct S1653Kfs mutation in *EYS* gene through in genome editing. **f**. Illustration of microhomology-mediated end-joining mutation replacement strategy. Genome of interest (GOI) with and without the S1653Kfs mutation are excised at the flanking gRNA target sites (gRNA-T; dotted line) from LCL genome and plasmid vector, respectively, by two gRNAs (1 and 4) and SaCas9. GOI without S1653Kfs mutation is inserted into the genome using MHA. gRNA-T, guide RNA target; PAM, protospacer adjacent motif; OHS, over-hanging sequence; NLS, nuclear localizing signal; pA, ploy A; U6, human U6 promoter.

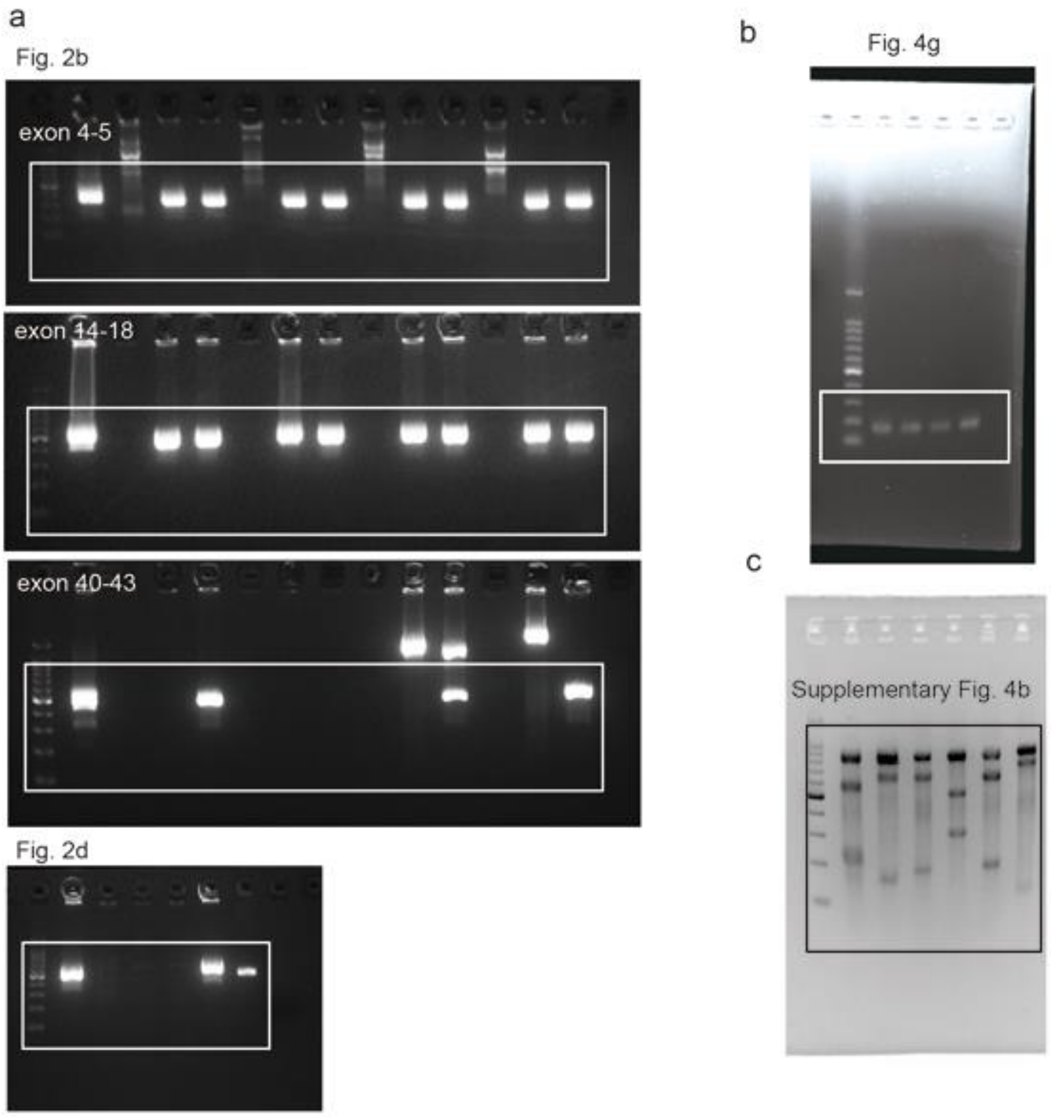

Supplementary Figure 5. Uncropped images of the gel electrophoresis presented in the main and supplementary figures.

a. Uncropped gels from Figure 2. **b**. Uncropped gel from Figure 4g. **c**. Unclopped gel from supplementary Figure 4b.

Gene_name	Genomic location	Chr	Coordinate	Ref	Alt	ToMMo AF	Odds ratio	P-value
NT5C1B-RDH14(dist=58909),LINC01376(dist=337974)	intergenic	2	18829755	A	T	0.073	0.128	2.70E-07
EGR4(dist=3282),ALMS1(dist=88775)	intergenic	2	73524111	G	GGT	ND	1.723	3.93E-06
CAMK2A	intronic	5	149610967	AAAT	A	ND	0.558	7.37E-06
EYS	intronic	6	65728469	C	T	ND	4.005	8.61E-10
DNAAF5	intronic	7	791207	ATT	A	ND	1.621	7.71E-06
STK31(dist=421431),NPY(dist=30246)	intergenic	7	24293561	G	A	0.140	1.914	8.27E-06
SEMA3A(dist=231370),LOC101927378(dist=106205)	intergenic	7	84055587	T	TATAG	ND	0.494	9.55E-06
NOC3L	exonic (:c.A1415C:p.E472A)	10	96104665	T	G	0.305	0.632	9.12E-06

Supplementary Table 1. List of candidate loci at $P < 1.0 \times 10^{-5}$ after 1st GWAS

Chr, chromosome; Ref, reference sequence; Alt, alternative sequence; AF, allele frequency.

Gene_name	Genomic location	Chr	Coordinate	Ref	Alt	ToMMo AF	Odds ratio	*P*-value
CPNE4	intronic	3	131630949	G	GGTTTTA	0.2976	0.480	5.83E-06
EYS	intronic	6	64595973	G	A	0.5567	0.480	1.93E-07

Supplementary Table 2. List of candidate loci at $P < 1.0 \times 10^{-5}$ after 2nd GWAS

Chr, chromosome; Ref, reference sequence; Alt, alternative sequence; AF, allele frequency.

Gene_name	Genomic location	Chr	Coordinate	Ref	Alt	ToMMo AF	Odds ratio	P-value
LINC01767(dist=29496),PLPP3(dist=49285)	intergenic	1	56911134	A	T	0.0263	1.97	5.73E-06
GS1-279B7.1(dist=108903),LINC01350(dist=114438)	intergenic	1	185413074	C	T	0.3195	0.68	6.67E-06
HAAO(dist=134357),LINC01819(dist=100884)	intergenic	2	43154108	G	A	0.3156	0.63	1.90E-06
LINC02233(dist=357406),FSTL5(dist=1248702)	intergenic	4	161056342	C	CA	ND	0.59	7.89E-07
STC2	intronic	5	172750120	C	A	0.3541	1.60	8.30E-06
EYS	intronic	6	65700352	A	G	0.0414	3.95	1.18E-13
C8orf87(dist=113319),LINC00535(dist=66297)	intergenic	8	94292398	C	A	0.0001	5.18	1.42E-06
XKR4	intronic	8	56130779	G	T	0.0042	4.00	5.46E-06
SGCZ	intronic	8	14999045	G	C	0.0693	2.12	9.28E-06
LINC02451	ncRNA_intronic	12	43047088	G	T	0.3846	0.66	2.11E-06
OAS2(dist=19943),DTX1(dist=26191)	intergenic	12	113469471	G	A	0.4733	0.71	7.76E-06
ITPR2	intronic	12	26860830	CA	C	ND	0.65	9.88E-06
RPL28(NM_001136134:c.*1285A>G, NM_000991:c.*1169A>G)	UTR3	19	55900869	A	G	0.8789	0.55	9.43E-06

Supplementary Table 3. List of candidate loci at *P* < 1.0 x10^{-5} after meta GWAS

Chr, chromosome; Ref, reference sequence; Alt, alternative sequence; AF, allele frequency.

R^2 values

	G843E (chr6:65622490)	S1653fs (chr6:65300802)	G2186E (chr6:64791763)	Y2935X (chr6:64431122)
Peak 1 (chr6:65700352)	**0.6817**	0.0133	0.0021	< 0.0001
Peak 2 (chr6:64602534)	< 0.0001	0.0004	< 0.0001	0.0031
Peak 3 (chr6:65320870)	0.0186	**0.7750**	0.0001	0.0011

D' values

	G843E (chr6:65622490)	S1653fs (chr6:65300802)	G2186E (chr6:64791763)	Y2935X (chr6:64431122)
Peak 1 (chr6:65700352)	**0.9131**	0.1730	0.1541	nd
Peak 2 (chr6:64602534)	nd	0.1181	0.0021	0.2691
Peak 3 (chr6:65320870)	0.1978	**0.9397**	1.0000	1.0000

Supplementary Table 4. Evaluation of linkage disequilibrium of top SNPs for *EYS* peaks in retinitis pigmentosa patients

Primer	Sequence (5'>3')	Use
Ex4-F	GTGGCTGAGTGTTGGGACAC	RT-PCR (Fig. 2)
Ex5-R	ATGGAAACAGACATGTGGTTGA	RT-PCR (Fig. 2)
Ex14-F	GGACATTGATGACTGCATCC	RT-PCR (Fig. 2)
Ex18-R	AGAGATCCAGAAAACCCAGG	RT-PCR (Fig. 2)
Ex40-F	GTTGGCCAGTGTCATGCTTC	RT-PCR (Fig. 2)
Ex43-R	CGCCAAGGTTGTAGCGAAGT	RT-PCR (Fig. 2)
ATGMO	CTCATGTTTGTCTTGGCTCGACTGG	Morpholino
SPMO1	TTGACTTACCCTTAAATCCTGGTG	Morpholino
SPMO2	AAAGTTCCTTCACTGTGAATGGAGC	Morpholino
SPMO3	CAGAGCAGTTGACACCTATGATGTG	Morpholino
Standard control	CCTCTTACCTCAGTTACAATTTATA	Morpholino
Eys201F	ACGTTGACGGATGCTATGAGCAG	RT/qRT-PCR (Fig. 4)
Eys201R	AGCAAGCTCCTTCTTTGCACC	RT/qRT-PCR (Fig. 4)
DrGAPDHF1	TCACACCAAGTGTCAGGACG	RT/qRT-PCR (Fig. 4)
DrGAPDHR1	CGCCTTCTGCCTTAACCTCA	RT/qRT-PCR (Fig. 4)

Supplementary Table 5 Primers used for RT-PCR
