# Supplementary material for: A hypomorphic variant in EYS detected by genome-wide association study contributes toward retinitis pigmentosa
Source: Commun Biol. 2021 Jan 29;4:140. doi: 10.1038/s42003-021-01662-9 (PMC7846782; doi:10.1038/s42003-021-01662-9)
Supplement: Supplementary file 3 — Description of Additional Supplementary Files [file 42003_2021_1662_MOESM3_ESM.pdf]

Description of Additional Supplementary Files

File name: Supplementary Data 1

Description: Source data for Figure 4h and 4q. For measuring eys mRNA expression by quantitative RT PCR, Ct values for eys were normalized against those for gapdh. Data for SPMO1, SPMO2, and SPMO3 were further normalized against that for ContMO (Figure 4h). Number of cells with mislocalized rhodopsin within a retinal section was counted (Figure 4q).
