# Supplementary material for: A hypomorphic variant in EYS detected by genome-wide association study contributes toward retinitis pigmentosa
Source: Commun Biol. 2021 Jan 29;4:140. doi: 10.1038/s42003-021-01662-9 (PMC7846782; doi:10.1038/s42003-021-01662-9)
Supplement: Supplementary file 5 — Reporting Summary [file 42003_2021_1662_MOESM5_ESM.pdf]

nature research

Corresponding author(s): Koji Nishiguchi

Last updated by author(s): Nov 16, 2020

Reporting Summary

Nature Research wishes to improve the reproducibility of the work that we publish. This form provides structure for consistency and transparency in reporting. For further information on Nature Research policies, see Authors & Referees and the Editorial Policy Checklist .

Statistics

For all statistical analyses, confirm that the following items are present in the figure legend, table legend, main text, or Methods section.

n/a	Confirmed	
[ ]	[x]	The exact sample size (*n*) for each experimental group/condition, given as a discrete number and unit of measurement
[ ]	[x]	A statement on whether measurements were taken from distinct samples or whether the same sample was measured repeatedly
[ ]	[x]	The statistical test(s) used AND whether they are one- or two-sided<br>*Only common tests should be described solely by name; describe more complex techniques in the Methods section.*
[ ]	[x]	A description of all covariates tested
[ ]	[x]	A description of any assumptions or corrections, such as tests of normality and adjustment for multiple comparisons
[ ]	[x]	A full description of the statistical parameters including central tendency (e.g. means) or other basic estimates (e.g. regression coefficient) AND variation (e.g. standard deviation) or associated estimates of uncertainty (e.g. confidence intervals)
[x]	[ ]	For null hypothesis testing, the test statistic (e.g. *F*, *t*, *r*) with confidence intervals, effect sizes, degrees of freedom and *P* value noted<br>*Give P values as exact values whenever suitable.*
[x]	[ ]	For Bayesian analysis, information on the choice of priors and Markov chain Monte Carlo settings
[x]	[ ]	For hierarchical and complex designs, identification of the appropriate level for tests and full reporting of outcomes
[x]	[ ]	Estimates of effect sizes (e.g. Cohen's *d*, Pearson's *r*), indicating how they were calculated

Our web collection on statistics for biologists contains articles on many of the points above.

Software and code

Policy information about availability of computer code

Data collection	PCA analysis was carried out using the 1000 Genomes Project (Phase 3) and PLINK software (ver.1.90).<br>SHAPEIT (ver.2.12) was used for phasing the obtained genotype information.<br>minimac3 (ver.2.0.1) was used for genotype imputations.<br>GWAS was carried out using P-LINK (ver.1.90).<br>Meta-GWAS was done using METAL (ver.2011-03-25).<br>The sequenced reads were aligned to the human reference genome using BWA-mem (ver. 0.7.17).<br>PCR duplicate reads were marked using Picard tools (ver. 2.17.8).<br>SNVs and short insertions and deletions were called, using GATK (ver. 4.1.2.0)<br>Primer3 (ver. 0.4.0) was used to design primers.<br>A sample size calculator was used (https://www.stat.ubc.ca/).<br>Sequences were aligned by ClustalW (https://clustalw.ddbj.nig.ac.jp/)
Data analysis	Statistical analysis was performed by JMP Pro 13.10 (SAS Institute, Cary, NC).

For manuscripts utilizing custom algorithms or software that are central to the research but not yet described in published literature, software must be made available to editors/reviewers. We strongly encourage code deposition in a community repository (e.g. GitHub). See the Nature Research guidelines for submitting code & software for further information.

Data

Policy information about availability of data

All manuscripts must include a data availability statement. This statement should provide the following information, where applicable:

- Accession codes, unique identifiers, or web links for publicly available datasets
- A list of figures that have associated raw data
- A description of any restrictions on data availability

Raw data used during and/or analyzed for all Figures and Tables are available from the authors within the limits of the Institutional ethical and research approvals, upon a reasonable request.

Field-specific reporting

Please select the one below that is the best fit for your research. If you are not sure, read the appropriate sections before making your selection.

[x] Life sciences [ ] Behavioural & social sciences [ ] Ecological, evolutionary & environmental sciences

For a reference copy of the document with all sections, see nature.com/documents/nr-reporting-summary-flat.pdf

Life sciences study design

All studies must disclose on these points even when the disclosure is negative.

Sample size	Since it was very difficult to estimate the outcome initially because we could not find a GWAS targeting recessive Mendelian disorders, we estimated the size of the second GWAS based on the results of the first GWAS assuming that meta-GWAS was to be performed. The first GWAS was carried out using all the samples available at that time. Sample sizes for the second GWAS were calculated so that top 5 signals would reach statistical significance using on-line sample size calculator (https://www.stat.ubc.ca/) adopting a two-sided alpha-level of 0.05, 80% power. However, size was eventually restricted by the availability of the samples because the disease studied was a rare disease with a prevalence of 1 in 4000.
Data exclusions	As for GWAS, samples with poor data quality, related samples, racial outliers were excluded following standard procedures as described in detail in the Methods.
Replication	Data for Figure 2 has been confirmed to be reproducible by at least 3 independent assays. For Figure 4, 3 blinded observers independently analyzed the data and showed similar results.
Randomization	Treatment for zebrafish was randomly allocated.
Blinding	For analysis related to Figure 2, samples were not blinded. As for Figure 4, observers were blinded to the treated conditions.

Reporting for specific materials, systems and methods

We require information from authors about some types of materials, experimental systems and methods used in many studies. Here, indicate whether each material, system or method listed is relevant to your study. If you are not sure if a list item applies to your research, read the appropriate section before selecting a response.

Materials & experimental systems

n/a	Involved in the study	
[ ]	[x]	Antibodies
[ ]	[x]	Eukaryotic cell lines
[x]	[ ]	Palaeontology
[ ]	[x]	Animals and other organisms
[ ]	[x]	Human research participants
[x]	[ ]	Clinical data

Methods

n/a	Involved in the study	
[x]	[ ]	ChIP-seq
[x]	[ ]	Flow cytometry
[x]	[ ]	MRI-based neuroimaging

Antibodies

Antibodies used	Rabbit polyclonal anti-Eys antibody (1:200, Novus Biologicals, Centennial, Colorado, USA)<br>Rabbit polyclonal anti-rhodopsin (bovine) antibody (1:500, Abcam, Cambridge United Kingdom)<br>Mouse monoclonal [6-11B-1] anti-alpha Tubulin (acetyl K40) antibody (1:200, Abcam, Cambridge United Kingdom)<br>Alexa Fluor 594-conjugated IgG antibodies (1:500, Jackson ImmunoResearch, West Grove, Pennsylvania, U.S.A.)
Validation	The antibodies have been validated by their manufacturers as described on the company website.

Eukaryotic cell lines

Policy information about cell lines

Cell line source(s)	LCL: Lymphocytes were transformed with the Epstein-Barr virus at a core facility run by Tokyo Medical Dental University.
Authentication	The origin of the LCL cells were identified as patients by partial sequencing of the genome.
Mycoplasma contamination	The cells used were mycoplasma free. We test cells routinely for mycoplasma contamination.
Commonly misidentified lines (See ICLAC register)	No commonly misidentified cell lines were used.

Animals and other organisms

Policy information about studies involving animals; ARRIVE guidelines recommended for reporting animal research

Laboratory animals	Zebrafish (Danio rerio), AB strain
Wild animals	No wild animals were used in this study.
Field-collected samples	Study did not involve specimens collected from the field.
Ethics oversight	All experimental procedures were conducted after approval by the related committees, including animal ethics committee, for the animal experiments at Osaka University Graduate School of Medicine.

Note that full information on the approval of the study protocol must also be provided in the manuscript.

Human research participants

Policy information about studies involving human research participants

Population characteristics	All patients and controls used for analyses were consistent with their being Japanese origin as described in the Methods.
Recruitment	The majority of the patients were recruited through a genetic screening project hosted by the Japan Retinitis Pigmentosa Registry Project (JRPRP) in which 83 genes associated with RP were analyzed by targeted re-sequencing (reference 11). The remaining patients were recruited from Tohoku University Hospital. Most of the unaffected controls, who were ruled out for RP with a fundus examination, were recruited at Tohoku University Hospital and its affiliated hospitals. The remaining control samples from subjects with no documented history of ocular disease were purchased from the National Institutes of Biomedical Innovation, Health and Nutrition (https://bioresource.nibiohn.go.jp/).
Ethics oversight	The study was initiated after ethical approvals were granted by the Institutional Review Boards of Kyushu University Hospital, Tohoku University Hospital Yuko Wada Eye Clinic, Nagoya University Hospital, and Juntendo University Hospital,.

Note that full information on the approval of the study protocol must also be provided in the manuscript.
